# Transmembrane Peptides as Inhibitors of Protein-Protein Interactions: An Efficient Strategy to Target Cancer Cells?

**DOI:** 10.3389/fonc.2020.00519

**Published:** 2020-04-15

**Authors:** Camille Albrecht, Aline Appert-Collin, Dominique Bagnard, Sébastien Blaise, Béatrice Romier-Crouzet, Roman G. Efremov, Hervé Sartelet, Laurent Duca, Pascal Maurice, Amar Bennasroune

**Affiliations:** ^1^Université de Reims Champagne-Ardenne, Reims, France; ^2^CNRS UMR 7369, Matrice Extracellulaire et Dynamique Cellulaire, MEDyC, Reims, France; ^3^Université de Strasbourg, Strasbourg, France; ^4^INSERM U1119 Biopathologie de la Myéline, Neuroprotection et Stratégies Thérapeutiques, Labex Medalis, Fédération de Médecine Translationnelle de Strasbourg, Strasbourg, France; ^5^M. M. Shemyakin and Yu. A. Ovchinnikov Institute of Bioorganic Chemistry, Russian Academy of Sciences, Moscow, Russia; ^6^Higher School of Economics, Moscow, Russia

**Keywords:** transmembrane peptides, protein-protein interaction, transmembrane protein dimerization inhibition, cancers, peptide delivery strategy

## Abstract

Cellular functions are regulated by extracellular signals such as hormones, neurotransmitters, matrix ligands, and other chemical or physical stimuli. Ligand binding on its transmembrane receptor induced cell signaling and the recruitment of several interacting partners to the plasma membrane. Nowadays, it is well-established that the transmembrane domain is not only an anchor of these receptors to the membrane, but it also plays a key role in receptor dimerization and activation. Indeed, interactions between transmembrane helices are associated with specific biological activity of the proteins as cell migration, proliferation, or differentiation. Overexpression or constitutive dimerization (due notably to mutations) of these transmembrane receptors are involved in several physiopathological contexts as cancers. The transmembrane domain of tyrosine kinase receptors as ErbB family proteins (implicated in several cancers as HER2 in breast cancer) or other receptors as Neuropilins has been described these last years as a target to inhibit their dimerization/activation using several strategies. In this review, we will focus on the strategy which consists in using peptides to disturb in a specific manner the interactions between transmembrane domains and the signaling pathways (induced by ligand binding) of these receptors involved in cancer. This approach can be extended to inhibit other transmembrane protein dimerization as neuraminidase-1 (the catalytic subunit of elastin receptor complex), Discoidin Domain Receptor 1 (a tyrosine kinase receptor activated by type I collagen) or G-protein coupled receptors (GPCRs) which are involved in cancer processes.

## Introduction

Membrane proteins are defined as proteins found in cell membrane either at the surface or on intracellular organelles and represent around 30% of all eukaryotes and prokaryotes proteins. Membrane proteins are classified as transmembrane (TM) or peripheral proteins. Their membrane-spanning domains are described to be structured as β-sheets in bacteria and mitochondria or essentially as α-helices (1, 2). The TM proteins (single or multi-pass membrane proteins) are involved in several cellular processes such as cell signaling, cell-cell communication, transport, energy transduction, and activation of enzymes which induce several functions like cell proliferation, migration, and differentiation. These cellular responses are induced by external stimuli and mediated by signaling pathways activated by membrane receptors associated with a large panel of proteins constituting complex signal networks (3, 4). Their role in cellular and physiological responses, and consequently in pathologies associated with their dysfunctions, lead researchers to develop several strategies to target these membrane proteins.

Activation of membrane receptors occurs most of the time by dimerization or oligomerization of these single-pass proteins in cell membranes and cumulative data underline the role of TM/TM domain interactions during the formation of these receptor complexes (5–8). Nowadays, it is well-established that the TM domain plays a key role in receptor dimerization and activation (9). Indeed, interactions between TM helices are associated with specific biological activity of these proteins. Overexpression or constitutive dimerization (due notably to mutations) of these TM receptors are involved in several physiopathological contexts as cancers. The TM domain of receptor tyrosine kinase (RTK) as ErbB family proteins (associated with several cancers) or other receptors as Neuropilin and G-protein coupled receptors (GPCRs) has been described these last years as putative targets to inhibit their dimerization/activation using several strategies. In this review, we will focus on the history of a strategy which consists of using peptides to disturb in a specific manner the interactions between TM domains and the signaling pathways induced by ligand binding of ErbB receptors and Neuropilins. This approach can be extended to inhibit other TM protein dimerization such as neuraminidase-1 (Neu-1, the catalytic subunit of elastin receptor complex), DDR1 (Discoidin Domain Receptor 1, a RTK activated by type I collagen) and GPCRs which are involved in cancer processes.

## Target Receptor Transmembrane Domain: TM Peptides Strategy

**Figure 1:**
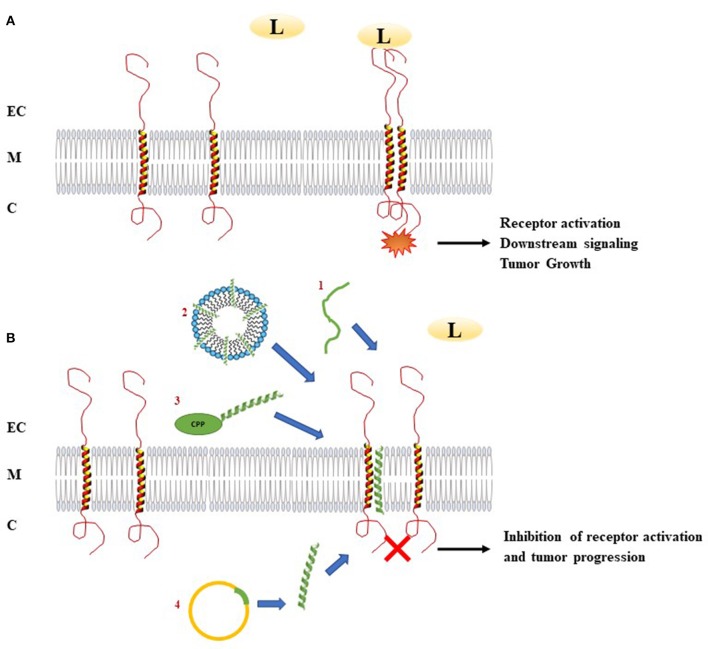
Mechanism of action of transmembrane peptides to inhibit receptor dimerization. **(A)** Ligand binding induces dimerization and activation of receptors. This activation leads to downstream signaling activation. In the case of abnormal activation, receptor dimerization can be associated with pathological processes as tumor growth. **(B)** Introduction of TM hydrophobic peptides which mimic the TM segments of membrane proteins involved in cancers can disturb the dimerization of these receptors. In order to deliver TM peptides, several methods could be used: (1) acidity-Triggered Rational Membrane (ATRAM) peptide, (2) delivery of the peptide by detergent micelles, (3) delivery of the peptide using cell penetrating peptide, (4) plasmid encoding TM peptide. C, Cytoplasm; CPP, Cell penetrating peptide; EC, Extracellular environment; L, Ligand; M, Membrane; TM peptide, Transmembrane peptide.

Most of the membrane receptors involved in cancer are single pass membrane receptors including RTKs, the integrins and the cytokine receptors. From HER2/ErbB2 (10), being the origin of one of the first targeted therapy, to for example VEGFR (11), RTKs are crucial players controlling abnormal cell proliferation, migration, or tumor angiogenesis. Consistently, several approaches had been developed to block them in order to fight cancer progression. Classical strategies using small molecules or blocking function antibodies showed tremendous therapeutic effects that contributed to significant increase of patient survival or remission in many different types of cancer. However, these targeted strategies still suffer from major hurdles such as resistance or compensatory mechanisms as exemplified for EGFR inhibitors (12) adding to often severe side effects of the drugs (13). Facing the need of developing new drugs potentially addressing these challenges, conceptual studies moved from extracellular or intracellular domains of membrane receptors to explore whether the TM domain could be an alternative solution to current drug design. Indeed, TM domains contribute in the dimerization of membrane receptors and their role in multimerization to form dynamic receptor platforms ensures complex biological functions in response to the diversity of ligands. Involvement of TM domains in these processes thus defines a totally virgin territory to design new drugs which may meets the eyes for more efficient and less toxic therapeutic compounds. As the TM domains of a multitude of membrane proteins are directly involved in receptor dimerization and activation, several strategies using short hydrophobic peptides have been developed as tools to target specifically the corresponding receptor activation (Figure 1). This part will describe the main results concerning the targeting of several membrane proteins involved in cancers by TM hydrophobic peptides which mimic the TM segments of these receptors.

### ErbB Receptor TM Domains as Targets in Cancer

Concerning receptors activated by dimerization, the case of RTKs, notably ErbB receptors, is among the more described. The ErbB family receptors include epidermal growth factor receptor (EGFR/ErbB1), ErbB2, ErbB3, and ErbB4 which are expressed ubiquitously in epithelial, mesenchymal, cardiac and neuronal cells. These receptors are associated with several cellular processes—as proliferation, survival, and angiogenesis—and are often dysregulated in cancers (14). In most cases, the binding of growth factors to the extracellular region of these receptors provokes their dimerization and their activation (14–16).

Several studies have highlighted a major role for interactions between TM domains and their importance in membrane protein structure, function and assembly. Furthermore, mutations in these TM domains are often associated with numerous pathological contexts (5–8, 17). Although it was initially thought that the TM domain of RTKs as ErbB receptors was a passive anchor to the membrane, it is now well-established that it plays a key role in protein dimerization. Indeed, previous studies have shown that TM domain of these proteins are able to self-assembly and induce biological activity. For instance, Val^644^ to Glu mutation within the TM domain of ErbB2/Neu is associated with an uncontrolled activation of this RTK leading to glioblastomas in rats (18). Moreover, Gardin et al. (19) showed that changing the TM domain of the insulin receptor (IR) with the highly dimerizing TM domain of glycophorin A is associated with an inhibition of insulin-induced receptor kinase activity. Other works have shown that homodimerization of EGFR/ErbB1 receptors is linked to interactions between their TM domains (20) and that TM domains of ErbB receptor family members can spontaneously homodimerize in cell membranes (21–23). Furthermore, sequence motifs have been reported to mediate TM domain interactions: the GxxxG motifs (x = any amino acid) or GxxxG-like motifs (a consensus sequence that has been extended to SmallxxxSmall sequences where small amino acids are glycine, alanine, or serine). These sequences are very frequent in TM helix and represent the core of dimerization interface (5, 24).

As interactions between TM segments occur during receptor dimerization, several studies have been carried out to evaluate if introduction in the membrane of peptides with homologous sequences—corresponding to the TM domain—could act as competitors of the dimerization and thus disturb the cancer involved RTK activity. Lofts et al. (25) showed for the first time that expression of TM sequence of the rat neu/ErbB2 receptor could inhibit cell growth of mutant-transformed NIH3T3 cells. As this work did not include characterization of receptor activation, several subsequent studies demonstrated that TM domain-derived peptides are able to interfere with dimerization of ErbB2 receptors in whole cells. Indeed, Bennasroune et al. (26) demonstrated in human cancer cells which overexpress EGF or ErbB2 receptors that TM peptides are able to specifically inhibit the autophosphorylation and the signaling pathway of their cognate receptor. These results were obtained using two strategies: the first one consisted of using expression vectors encoding fusion TM peptides and the second one consisted of incubating cells with chemically synthesized peptides. This study was extended and confirmed by the same research group who demonstrated that in cells overexpressing chimeric IR (where the TM domain has been replaced by that of EGFR or a mutated ErbB2 domain), TM peptides can inhibit specifically the autophosphorylation and the signaling pathway of IR with the corresponding domain (27).

Thereafter, few studies using TM peptide as tools to target specifically protein dimerization have been realized *in vivo*. Concerning RTK, Arpel et al. (28) showed that small peptides interfering with the TM domain of ErbB2 inhibit breast tumor growth and metastasis when used at micromolar concentrations in a mouse model of breast cancer. Thus, even if there was a disdain toward the use of peptides as a strategy to inhibit protein-protein interaction, this technique has been extended to target other membrane proteins involved in cancers.

### Neuropilin and Plexin TM Domain as Targets in Cancers

Other membrane receptors such as Neuropilins (NRP1) or Plexins are also important regulators of cancer progression through signaling pathways involving actin cytoskeleton remodeling (29). NRP1 TM domain which contributes to the dimerization of the receptor was shown mandatory to trigger Sema3A-dependent cancer cell migration (30). It turns out to be an efficient strategy to limit glioblastoma (31) or breast cancer (32) growth *in vivo*. These studies used a peptide mimicking the TM domain of NRP1 to interfere with the dimerization by direct competition for binding with the natural TM domain of the membrane receptor. To circumvent the hydrophobic nature of the peptides which may preclude their use, the decoy peptides were solubilized in micelles of Lithium Dodecyl Sulfate favoring delivery to the membrane both *in vitro* or *in vivo*. Biological or therapeutic effects were observed with low dose of the peptide (in the range of 1 μg/kg *in vivo*, three times per week after intraperitoneal injection) and showed remarkable tolerance. The same strategy of mimetic peptide was also used to target HER2 (ErB2) in the context of metastatic breast cancer as described above (28). In the same line, the interference of Plexin-A1 heterodimerization with a peptide mimicking the natural sequence of this Rho-GTPase activating receptor exhibited anti-angiogenic effects in models of brain tumors and glioblastoma cancer stem cells growth (33). More recently the same Plexin-A1 targeting peptide was shown as an efficient tool to circumvent the Sema3A molecular barrier blocking the remyelination process in the context of demyelinating diseases (34). Because TM peptides interact with intra-membrane targets, they do not have the capacity to be used to selectively reach the cancer cells expressing the target. Rather, they exert their function as small molecules by a widespread distribution in the body. However, TM domain peptides can be combined with targeting moieties attached to nanocarriers to address this point and produce drugs with a more selective action on a given cell type (35). While the development of formulations compatible with a clinical use remains to be fully achieved, the recent development in the production of TM domain peptides with pH sensitive membrane interaction is opening interesting opportunities both in term of solubility or activity. The so-called acidity-triggered rational membrane (ATRAM) peptides demonstrated preferential membrane insertion in breast cancer cells and exhibited prolonged circulating time in the blood thanks to a reversible binding to serum albumin (36).

## Transmembrane Peptide Strategy Extended to Inhibit Other Transmembrane Protein Dimerization: Neu-1, DDR-1, and GPCRs as Putative Targets

The transmembrane peptide strategy can be extended to inhibit other TM protein dimerization. Even if several membrane proteins can be targeted by this approach, three examples will be described in this section: Neu-1, DDR1, and GPCRs which are involved in several cancer processes.

### Neu-1 TM Domain as a Potential Target in Cancers

Elastin degradation contributes to cancer progression (37). The interaction of cancer cells with elastin-derived peptides (EDP) induces mitogenic signals and a release of elastases that enhance further elastin degradation (38). Most of the biological effects of elastin degradation and EDP rely on the catalytic activity of Neu-1 activated upon the binding of EDP on the elastin receptor complex (ERC). This membrane heterotrimeric complex is composed of the elastin-binding protein (EBP), a spliced variant of the lysosomal β-galactosidase which interacts with EDP and tropoelastin, PPCA/Cathepsin A ensuring the integrity of the complex, and Neu-1 harboring a sialidase/neuraminidase activity (39). Neu-1 is a member of the sialidase family composed also of Neu-2, Neu-3, and Neu-4 (40). These exoglycosidases, widely distributed amongst species (41), remove terminal sialic acid residues from glycoproteins, glycolipids, and oligosaccharides.

Neu-1 regulates breast cancer cell proliferation and invasion. EDP enhance invasiveness of MDA-MB-231 cells by enhancing matrix metalloproteases (MMP) 2 and 14 activities (42). Moreover, an increase of elastolysis is correlated with severity of the disease. Clinical studies show that level of EDP in serum of patients is higher in patients with large tumor size (43). Blocking Neu-1 with oseltamivir phosphate (Tamiflu®) or a Neu-1 siRNA in mammary carcinoma cells, MCF-7, and MDA-MB-231 cell lines, inhibits cell growth (44). Additional studies also point out an inhibition of tumor neovascularization growth and metastasis under oseltamivir phosphate treatment in mouse model of breast cancer that mimics human triple-negative breast cancer (45). Furthermore, EDP induce an enhanced invasion of melanoma cells (46–48). Implication of EDP and Neu-1 in other cancer types has also been shown: Neu-1 is involved in the development of hepatocellular carcinoma (49) and ovarian cancer (50). Altogether, these data indicate that Neu-1 plays a key role in the development and the amplification of several cancers and can constitute a new target to slow down cancer progression.

Recent studies have identified two potential TM domains in the sequence of human Neu-1 protein (51). Dynamic molecular simulation studies underline that the TM domain 2 (TM2) is able to preserve a stable helical conformation and homodimerizes in membrane-mimicking environments. Further, molecular biology experiments show that Neu-1 sialidase activity is linked to its ability to homodimerize. Point mutations in the TM2 region of Neu-1 are able to inhibit homodimerization and its associated sialidase activity. Indeed, when EDP bind on EBP, two Neu-1 subunits homodimerize and generate a sialidase activity. Knowing that dimerization is required for its activity, interfering peptides targeting specifically TM2 domain of Neu-1 constitute novel key tools to selectively block Neu-1 activity and its linked biological effects in cancer.

### DDR-1 TM Domain as a Potential Target in Cancers

The DDR belongs to RTKs family and consists of two members, DDR1 and DDR2. They possess an extracellular discoidin homology domain and are activated by the most abundant component of tumor extracellular matrix, native triple-helical collagen (52, 53). The expression of DDR1 in several different types of human cancer including human esophageal (54), gastric cancer (55), glioma (56), breast cancer (57), lung cancer (58), suggests a function in tumor progression. After activation by collagen, DDRs play a role in cell adhesion, proliferation, migration, invasion, and DDR1/myosin dependent extracellular matrix remodeling (59, 60).

Both DDRs have the same domain architecture containing a conserved discoidin I domain in their N-terminal extracellular part which is responsible for collagen binding, a single-span TM domain, an unusually large cytosolic juxtamembrane domain, and a C-terminal tyrosine kinase domain. After collagen binding, conformational modifications of the receptors are associated with a slow but sustained self-phosphorylation compared to other RTKs whose activation is rapid after ligand binding (52, 53). DDR1 activation induces transphosphorylation at the juxtamembrane and kinase domains of adjacent dimers. Moreover, this phosphorylation requires specific contacts within the TM domains but not in the extracellular domain (61).

One of the notable features is that in the absence of ligand, the DDRs form stable, non-covalent dimers kept in an inactivated state via N-glycosylation at highly conserved N211 residue (62–64). Contacts between extracellular domains, cytoplasmic domains, and TM regions contribute to the dimerization process. However, interaction between the extracellular and cytoplasmic regions is not critical for dimerization. Nevertheless, a mutation in the leucine zipper motif of the TM segment results in dimerization disruption highlighting the importance of TM region in ligand independent dimerization of DDR1 (63). The isolated DDR1 and DDR2 TM helices interact very robustly, as detected in a bacterial TOXCAT reporter assay (63). In fact, the comparison by a systematic study of the self-interaction potential of all RTK TM domains shows that the DDR1 and DDR2 TM domains gave the strongest signal of all RTKs in this assay (65). Activation of DDR is induced by forming lateral clusters in the presence of collagen thereby phosphorylating the DDR dimers leading to activation thanks to specific TM domain interactions. These data strengthen a key role for the DDR1 TM domain in signaling. TM domain contacts may also be necessary for DDR1 clustering, with direct receptor-receptor interactions or another membrane protein domain (61). These data suggest that TM peptides could be an adequate strategy to target the TM domain of DDR and in particular the leucine zipper motif to inhibit DDR activation and then receptor autophosphorylation at multiple residues on its tyrosine kinase intracellular domain. This inhibition could have an important role considering the involvement of these receptors in cancer progression but also in collagen processing events that contribute to fibrosis.

### GPCR TM Domain as a Putative Target in Cancers

GPCRs are the largest class of membrane receptors and play crucial roles in virtually every physiological process. Over the past few decades, the idea that these seven TM helical domain receptors function as isolated monomeric receptors has been challenged by the accumulation of evidence for the formation of homo- and hetero-dimers, and higher order oligomers. Combined with x-ray structures, computational molecular modeling, and bioinformatic approaches, synthetic TM peptides targeting the TM domains of GPCRs have been shown to be powerful tools to help in identifying the dimer interface of GPCRs and to examine the functional importance of GPCR dimerization both *in vitro* and *in vivo*. For instance, receptor homodimerization and agonist-dependent signaling can be inhibited by a synthetic TM peptide targeting the TM domain VI for the β2-adrenergic (66), IV for the secretin (67), and V for the A2A adenosine (68) receptors. Interestingly, TM peptides are also able to disrupt heterodimerization. A prototypical GPCR heterodimer is the one formed by the A2A adenosine receptor (A2AR) and D2 dopamine receptor (D2R). In a recent study by Borroto-Escuela et al. (69), TM peptides corresponding to the TM domain IV and V of the A2AR were shown to block heterodimer interactions and to disrupt the allosteric effect of A2AR activation on D2R agonist binding. Thus, the use of TM peptides permitted to identify the dimer interface of GPCRs and to understand the functional role of their dimerization. As in recent years, several studies have shown the involvement of these receptors in different cancer types, as breast and prostate cancers, using TM peptides could also be a very interesting strategy to target GPCRs in these pathologies (70).

## Conclusion

Overall, it is now well-established that interactions between TM domains are specific and play a crucial role in many membrane receptor activations. Consequently, this observation has been exploited to develop TM peptides as specific inhibitors of dimerization/activation of several receptors involved in cancers as RTKs and Neuropilins. However, as TM peptides interact with intra-membrane receptors, they do not have the capacity to selectively target the cancer cells expressing the target. Indeed, they exert their function as small molecules by a widespread distribution in the organism. That's why the next step will be to combine TM peptides with targeting moieties attached to nanocarriers to ensure specific delivery and to produce anti-cancer drugs with a more selective action on a given cancer cell type.

## Author Contributions

CA, AA-C, DB, PM, and AB participated in writing the manuscript. SB, BR-C, RE, HS, and LD realized a careful reading of the manuscript (and corrections).

### Conflict of Interest

The authors declare that the research was conducted in the absence of any commercial or financial relationships that could be construed as a potential conflict of interest.

## Abbreviations

A2AR: A2A adenosine receptor
D2R: D2 dopamine receptor
DDR: Discoidin Domain Receptor
EBP: Elastin-binding protein
EDP: elastin-derived peptides
ERC: Elastin receptor complex
EGFR: Epidermal Growth Factor Receptor
GPCRs: G-protein coupled receptors
HER: Human epidermal growth factor receptor
MMP: Matrix metalloproteases
Neu-1: Neuraminidase-1
NRP1: Neuropilin
PPCA: Protective protein/cathepsin A
RTK: Receptor tyrosine kinase
TM: Transmembrane
VEGF: Vascular endothelial growth factor.
